# “I Won’t Let the Past Destroy Me”: A Narrative Analysis of Identity Repair and Nascent Post-traumatic Growth Among Former Child Soldiers in Colombia

**DOI:** 10.1177/13591045251387378

**Published:** 2025-10-22

**Authors:** Mathew H Charles

**Affiliations:** 1 Faculty of International, Political and Urban Studies, 25807 Universidad Del Rosario, Bogotá, Colombia

**Keywords:** Colombia, child soldiers, narrative identity repair, narrative analysis, post-traumatic growth, reintegration, creative auto/biography

## Abstract

This study explores how former child soldiers in Colombia narrate identity repair following their experiences of conflict. Twenty-five adolescents and young people (aged 14–19) participated in participatory life-history workshops using a creative auto/biography (CAB) method. A narrative analysis of over 200 first-person multimodal texts identified four recurring plots— the Struggler, Learner, Advocate, and Survivor—each reflecting distinct, non-sequential pathways of self-reconstruction, emotional processing, and social re-engagement. These plots show how young people reimagine self and negotiate belonging amid stigma, rupture, and transition. The study proposes a Narrrative Identity Repair Compass comprising six interrelated domains: narrative coherence, self-concept (self-image and self-worth), self-efficacy (capacity to act on and shape circumstance), temporal orientation, relational positioning, and cognitive processing (narrative work integral to change). This multidimensional model conceptualises identity repair as fluid, non-linear, and relational. Post-traumatic growth (PTG) defined here as positive psychological change emerging from the struggle with major adversity is reframed as a contingent possibility shaped by reflection, relationships, and creativity. This framework offers a developmentally appropriate and context-sensitive approach to understanding how former child soldiers reconstruct meaning, repair identity, and move toward psychosocial integration following experiences of armed violence.

## Plain Language Summary

Stories of Survival and Growth: Young People Rebuilding Themselves After War: This study explores how young people who were formerly recruited by armed groups in Colombia begin to heal and rebuild their sense of self after experiencing violence and trauma. Rather than focusing only on psychological symptoms or diagnoses, the research looks at how these young people tell their life stories:what they choose to remember, how they make sense of difficult experiences, and how they imagine their future. Using a creative method called Creative Auto/Biography(CAB), 25 participants wrote, drew, and reflected on their past, present, and future. The research analysed over 200 pieces of writing/illustration and other creative artefacts to understand how young people process trauma and express growth through storytelling. The study found that while not all participants showed signs of growth, many developed new ways of thinking about themselves and their lives. These changes were often fragile, incomplete, and coexisted with pain, but they also reflected hope, purpose, and transformation. The research identified four narrative plots or identity types: Strugglers, whose stories were marked by confusion and unresolved trauma; Learners, who showed new insight and growing self-awareness; Advocates, who used their experiences to help others and imagine new futures; Survivors, who developed more stable and integrated identities despite past harm. From these stories, the study developed a model with six areas of growth: self-worth, emotional expression, narrative coherence, relational connection, moral repair, and future orientation. These findings can help social workers, therapists, and reintegration programmes better understand where young people are in their healing journeys and how to support them. By listening to their stories and recognising growth as uneven, we can support young people not just to recover, but to reimagine who they want to become.

## Introduction

The psychological and physical harms experienced by former child soldiers are well documented, with elevated rates of PTSD, anxiety, and aggression (Bayer et al., 2007; Derluyn et al., 2004; Kohrt, 2007). Trauma-focused research has often framed these young people as ‘morally damaged’ (Wessells, 2006) or defined by ‘fragmented’ selves (Peréz-Sales, 2010). While such perspectives illuminate the profound impact of armed violence, they risk underplaying these youths’ capacity to actively engage in their own recovery. In contrast, this study foregrounds narrative identity repair (Adler, 2012) and how former child soldiers mobilise storytelling to reclaim coherence, reassert agency, and author new meanings after disruption.

This emphasis on self-authored recovery aligns with the concept of post-traumatic growth (PTG), which recognises that highly distressing experiences of violence may not only cause harm, but can also prompt reflection, re-evaluation and psychological development (Tedeschi & Calhoun, 1996, 2004). PTG constitutes positive psychological change emerging from the struggle with major adversity—in this case, the aftermath of violent conflict and the challenges of reintegration marked by stigma, mistrust, and limited opportunities for belonging and livelihood. It entails qualitative shifts in worldview, identity, and functioning that go beyond recovery or resilience (Tedeschi & Calhoun, 1996, 2004). While resilience refers to maintaining or regaining prior levels of functioning, PTG implies a transformation to new ways of thinking, feeling, and relating. It has become an influential construct, but the theoretical and methodological foundations of PTG remain contested. Despite its emphasis on change beyond restoration, critics argue that PTG is sometimes conflated with coping and note its cultural specificity and reliance on self-report measures vulnerable to recall bias and social desirability (Zoellner & Maercker, 2006; Hobfoll et al., 2007). In post-conflict settings such as former child soldiering, these concerns are amplified: reintegration often hinges on presenting socially valued change, and narratives of growth or moral repair may be shaped as much by strategic self-presentation as by personal reflection.

Although Tedeschi and Calhoun (2004) acknowledged that PTG is constructed through cognitive and emotional struggle, much research continues to treat it as a fixed outcome. This risks missing how growth is developed and reconstructed over time. Viewing PTG as an evolving process invites closer attention to the developmental dynamics—particularly in adolescence—that shape how growth is initiated, sustained, and reworked.

Adolescence is a period of heightened identity exploration, emotional reactivity, and social reorientation (Erikson, 1968; Steinberg, 2005). These developmental features can magnify trauma’s impact, but also open unique pathways to growth (Kilmer, 2014). Evidence suggests that children and adolescents can experience PTG in ways similar to adults, though developmental stage and social context shape how it unfolds (Meyerson et al., 2011).

The fluidity of the adolescent self-concept can enable adaptive narrative reconstruction, particularly when supported by relational, symbolic, and reflective processes. For former child soldiers, identity repair is conceptualised as a developmental process through which these young survivors of conflict reconstruct meaning, self-concept, and social positioning after violence. PTG is framed not as that process itself, but as one possible—though not inevitable—trajectory within it.

## Method

The study adopts a narrative analysis to explore the lived experiences of participants. Data were collected using the CAB (Creative Auto/Biography) method, a participatory life-history approach developed by the author through work with former child soldiers and young offenders. CAB supports the co-creation of first-person narratives using artistic forms such as writing, photography, video, and audio over extended periods.

Grounded in narrative inquiry, the method assumes that identity is constructed through storytelling (Bruner, 2004; McAdams, 2001; Polkinghorne, 1991). The process of ‘emplotment’ (Ricoeur, 1984) links life events into meaningful wholes, enabling sense-making and self-recognition. CAB provides a supportive, reflexive space to explore past, present, and future selves.

The material produced through the CAB method was intentionally multi-layered. Alongside narrative texts, participants created poems, lyrics, photographs, drawings, and symbolic imagery—forms integral to their storytelling processes. These artistic outputs enabled expression when words felt insufficient to convey emotional complexity.

### Participants

Twenty-five adolescents and young people aged 14–19 took part. Inclusion criteria were: (i) experience of child soldiering; (ii) absence of acute trauma symptoms (screened using the Davison Trauma Scale - DTS); and (iii) informed, voluntary participation. Participants were recruited through purposive, non-clinical sampling in partnership with local humanitarian organisations (*N* = 25). The DTS was used solely to exclude acute distress that could be exacerbated by participation, ensuring ethical engagement while still including a range of trauma histories representative of former child soldiers’ experiences. While this criterion may have excluded those with the most acute symptoms, the sample still reflected substantial trauma exposure, and the findings should be interpreted with this ethical safeguard in mind. Informed consent was obtained from all participants, with additional guardian or child protection approval where needed (Table 1).

**Table 1.** Participant Overview

	Participants (*N* = 25)
Gender (%)	
Male	68
Female	32
Age during research project	
M	15.8
SD	1.6
Age of recruitment	
M	14.3
SD	1.1
Mode of recruitment* (%)	
Forced	76
Voluntary	24
Days spent as child soldier	
M	345.2
SD	190.4
Role as child soldier (%)	
Combatant	52
Intelligence	16
Logistics	12
Sexual violence	12
Communications	4
Other (catering, coca harvesters, drug dealers, extortion collectors)	4
Days spent in reintegration programme	
M	178
SD	81.3

### Data Collection

Participants took part in ten two-day workshops over three months. Workshops scaffolded reflection and narrative development through creative activities including object elicitation, mapping, and storytelling. CAB is designed to yield ‘autobiographic vignettes’ rather than comprehensive life histories (Charles & Fowler-Watt, 2020). These fragments reveal emotional insights, turning points, and shifts in perspective, often emerging non-linearly across sessions.

### Data Analysis

Over 200 participant-generated texts were analysed using Crossley’s (2000) six-step narrative method operationalised as: (i) six-pass immersion for each text/image; (ii) extraction of salient content (emotionally charged references, symbolic motifs); (iii) coding imagery/metaphor; (iv) linking symbols and narrative shifts to life events; (v) cross-case comparison to identify shared tensions or divergences; and (vi) synthesis into thematic groupings. Following Attride-Stirling's (2001) network model, basic themes (single ideas/events eg. *loneliness*) were clustered into organising themes (patterned meanings across case eg. *disrupted belonging*) and integrated as global themes aligned to four plots—Struggler, Learner, Advocate, Survivor—each articulated through six inductively derived domains (Table 2).

**Table 2.** A Narrative Analysis of Former Child Soldiers’ Texts

Basic codes	Clustered organising themes	Narrative plot (Global themes)	Example quotes^a^	Example visuals
Enduring hardship, emotional withdrawal, staying quiet, focusing on rules, surviving violence, grief, loneliness, shame, traumatic memories, rejection by family, instability in shelters	Suppression as strategy; emotional disconnection; fragmented self; disrupted belonging; unresolved trauma	*Struggler* — low narrative coherence; negative self-concept; low self-efficacy; past-bound temporal orientation; isolated relational positioning; cognitive processing dominated by intrusive rumination	Sometimes I don’t know who I am anymore. You get used to not feeling. You just focus on not messing up	Self-portrait in shadow, face partly obscured; empty chair in corner of room; future drawn as weapons and bloodshed
Reflective conversations, education access, peer empathy, realising alternative futures; writing letters, drawing timelines, discussing shame or guilt, reframing vulnerability	Emergent insight; creative reflection; emotional risk; self-redefinition; imagining new roles	*Learner* — increasing narrative coherence; emerging positive self-concept; growing self-efficacy; future-oriented temporal orientation; connected relational positioning; cognitive processing marked by deliberate reflection	I Started seeing myself differently when I heard others say they felt the same. Writing helped me realise I was more than just what I did	Drawing of open book and sunrise; timeline with ‘past’ crossed out and ‘future’ drawn in bright colours
Mentorship, activism, helping others, storytelling, imagining a future with purpose; public speaking, representing others, community engagement, moral framing of past	Social contribution; identity through service; transforming pain into purpose; relational responsibility	*Advocate* — integrated narrative coherence; positive self-concept tied to contribution; high self-efficacy; future-focused temporal orientation; prosocial relational positioning; cognitive processing involving moral reframing and meaning-making	I Talk to the younger ones now—if I can change, they can too. Maybe what I went through can help someone else avoid it	Group mural of linked hands; speaker at community event; megaphone producing peace doves
Sustaining routines (school, family etc), managing ongoing symptoms, actively maintaining support networks, goal setting and ambition, reflection and meaning making	Stability through structure; balance of vulnerability and resilience; emotionally grounded self-control; perseverance with purpose	*Survivor* — Stable narrative coherence; secure self-concept; sustained self-efficacy; strongly future-oriented temporal orientation; selective but trusting relational positioning; cognitive processing focused on maintaining stability rather than revisiting trauma	I don’t talk about the past much, but I’ve got my plan. I Keep going. I don’t need applause. Just to keep moving forward. I won’t let the past destroy me	Road stretching into horizon; well-organised personal calendar; path through a forest; hands clasped within those of another

^a^Original Spanish translated into English by the author.

Initial codes and definitions were established collaboratively through joint review of a subset of data; once guidelines were agreed, coders worked independently to apply and refine these into basic and organising themes before reconciling differences to calculate agreement rates prior to consensus. Plot membership was assigned when at least three distinct contributions (text and/or visual) matched a plot’s defining features. This threshold was set a priori to ensure recurrent rather than incidental alignment; multiple memberships were retained when criteria were met for more than one plot.

Visual and multimodal materials were analysed alongside texts using interpretive visual methodologies (Rose, 2000; Levy, 2015), including compositional analysis, metaphor/symbol coding, temporal cue identification, and emotional tone assessment (Table 2). Convergence was confirmed when narrative and visual modes expressed the same organising or global theme; divergences were re-examined in subsequent coding rounds to explore whether they reflected complementary meanings, indicated the need for theme refinement, or revealed new interpretive possibilities.

The research team comprised the author and four trained undergraduate research assistants. Rigour was supported by double-coding 40 texts (20% of the dataset) for basic themes and plot assignments (discrepancies resolved through discussion), alongside reflexive journaling, member checking, and iterative team debriefs to co-construct meaning and mitigate social desirability effects.

## Results

The narrative analysis of participants’ contributions identified four recurring plots, illustrating divergent overlapping experiences of identity repair: the Struggler, Learner, Advocate, and Survivor (Table 2).

Table 3 shows an overview of the frequency of the plots identified. Each plot represents a different narrative position rather than sequential progression. Participants’ experiences were often multi-layered, therefore many of them were coded in multiple plots.

**Table 3.** Overview of the Plots and Their Frequency

Plot	Participants (*N* = 25)^a^
Struggler	4
Learner	7
Advocate	8
Survivor	6

^a^Participants’ texts could be linked to multiple plots.

Participants often moved between or inhabited more than one plot simultaneously, reflecting the non-linear nature of identity repair. This narrative hybridity reinforces how the four plots are not fixed stages or endpoints but rather flexible configurations of identity work.

While certain experiences—particularly sexual violence—were strongly gendered, participants’ responses to these experiences still aligned with the four narrative plots, underscoring how each one captures variability in meaning-making rather than fixed, gender-specific pathways. Furthermore, while each plot intersects with grief and loss through mourning of people, roles, and futures, they represent neither sequential grief stages nor uniform steps toward PTG.

### Plot 1: The Struggler (Fragmentation and Ambivalence)

Struggler narratives revealed the emotional and narrative complexity of recovery. These incoherent narratives reflected confusion, disorientation, and unresolved emotional weight. While some explored self-expression through imagery or dialogue, many remained unsure of their identity or future. These accounts challenge assumptions about linear progress, illustrating how repair can remain suspended or deeply conflicted, with resilience still out of reach.

### Plot 2: The Learner (Reflection and Redefinition)

Learner narratives centred on introspection and efforts to reconfigure identity. Participants confronted shame, guilt, and past harm, moving through cycles of emotional struggle and tentative insight. Vulnerability was reframed as a source of strength, with creative practices like writing or dialogue used to construct new self-understandings. Though uncertain, these stories showed a shift from survival to meaning-making.

### Plot 3: The Advocate (Social Transformation)

Advocate narratives portrayed a shift from individual survival to collective purpose. Participants reimagined past suffering as fuel for social action, becoming mentors, storytellers, or community leaders. These stories projected clarity of mission and a strong relational drive. Even where pain remained unresolved, participants used contribution as a vehicle for self-renewal—indicating transformation not only in role but in worldview. Growth, here, involved more than bouncing back; it reflected a reorientation toward others grounded in moral agency. However, for some, the advocate role risked masking unresolved pain, offering external recognition without full emotional integration.

### Plot 4: The Survivor (Integration through Endurance)

The Survivor plot represented the most integrated form of personal growth. These narratives emphasised perseverance, emotional regulation, and the gradual reconstruction of life after conflict. Turning points led to self-control, future orientation, and selective trust. Survivors drew on inner strength to rebuild with purpose and clarity.

While age was not the focus of analysis, narrative trends suggested that older participants (aged 17–19) more often expressed elements of the Advocate or Survivor identities, whereas younger adolescents tended to align more closely with Learner or Struggler plots. This may reflect developmental differences in self-reflection, emotional regulation, and future orientation across adolescence (McLean et al., 2010). However, these trends were not uniform or prescriptive, and movement between plots occurred in multiple directions as meaning evolved.

### Evidence of Growth

The Learner, Advocate, and Survivor plots illustrate progressive yet still-unfinished shifts in identity and meaning-making beyond resilience or coping, reflecting how participants were actively reconstructing their sense of self, purpose, and possibility. Each plot contained ‘growth elements’ of PTG inventories (Cryder et al., 2006; Tedeschi & Calhoun, 1996). While not applied diagnostically, the typology captures PTG in formation: cognitive–emotional ‘expansions and developments’ in self and world (Janoff-Bulman, 2014, p. 82). This nascent growth was bound to a reconfiguration of temporal identity, as past suffering was linked to tentative future-oriented selves with renewed purpose (Table 4).

**Table 4.** PTG Among Former Child Soldiers

PTGI Growth elements	Struggler	Learner	Advocate	Survivor
Personal strength	Low: Confusion, shame, and emotional overwhelm limit access to inner strength	Moderate: Emerging strength through vulnerability and self-exploration	Strong: Confidence and agency expressed through prosocial roles	Very strong: Emotional regulation and perseverance in the face of adversity
Relating to others	Low: Mistrust and isolation dominate relational world	Moderate: Tentative efforts to build trust and connection	Strong: Relational commitment through helping others	Moderate to strong: Selective trust, emotionally grounded relationships
New possibilities	Low: Unclear or blocked future vision	Moderate: Emerging exploration of new roles and paths	Strong: Clear purpose and future orientation through social engagement	Strong: Stable, realistic future planning based on inner capacity
Appreciation of life	Low: Unresolved pain overshadows appreciation	Moderate to strong: Reflection fosters gratitude and renewed meaning	Strong: Suffering reframed through moral clarity and contribution	Moderate: Grounded in a deep sense of having endured
Spiritual/Existential change	Minimal: Existential confusion or disconnection	Some: Growing reflection on values, guilt, and purpose	Strong: Defined moral vision and sense of mission	Moderate: Pragmatic reflection, limited spiritual engagement

Conversely, the Struggler plot revealed the limits of growth. Participants in this group conveyed disorientation, pain, and narrative fragmentation. Rather than refuting PTG, these stories show that growth is not inevitable nor linear, and that recovery may be slow, contradictory or incomplete. In this sense, PTG is best understood as a fragile, uneven and contingent process.

### Cross-Cutting Themes: Settings, Characters, and Epiphanies

The four narrative plots were shaped by cross-cutting elements that influenced narrative direction and depth. These included the settings in which participants lived, the relationships that surrounded them, and the moments of personal insight or epiphanies that reoriented their sense of self. Together, these factors acted as either *enabling* or *undermining* influences, shaping whether participants were able to move toward transformation or remained caught in ongoing struggle.

### Settings: Institutional, Social, and Symbolic Spaces

Four recurring settings emerged: the reintegration centre, the school, the home, and the community. These were not passive backdrops but active elements in meaning-making, capable of prompting emotional reflection or reinforcing stigma (Table 5).

**Table 5.** Narrative Settings and Their Frequency

Plot	Participants (N)^a^	Enabling (N)	Undermining (N)
Reintegration centre	23	12	11
School	18	12	6
Home	14	5	9
Community	9	2	7

^a^Participants’ texts could be linked to multiple settigs (*N = 25*).

The reintegration centre was often experienced ambivalently—as a space of both control and safety. Its structured routines and psychosocial support provided some participants with early opportunities to reflect and rebuild.

Schools offered normalcy but also the risk of judgment, making them sites of both belonging and retraumatisation.

The home, though not physically returned to during reintegration, remained a powerful symbolic setting. For some, it evoked love, memory, or longing; for others, it was associated with neglect, violence, or complicity in their recruitment. As such, the home functioned as an affective landscape tied to both early vulnerability and ongoing desire for care.

The community—particularly urban neighbourhoods where participants were placed—could be disorienting and unsafe. Many came from rural areas and experienced reintegration into cities as alienating and hostile (Denov & Marchand, 2014). However, some found support in local initiatives such as church groups or artistic collectives, which offered opportunities for connection without directly confronting past trauma.

### Characters: Present and Absent Figures

Narratives were also shaped by the presence - and absence - of key figures. Relationships with mentors, family members, and peers often played a pivotal role in recovery, while missing or harmful figures left emotional voids that complicated meaning-making (Table 6).

**Table 6.** Characters and Their Frequency

Plot	Participants (N)^a^	Enabling (N)	Undermining (N)
Mentors	25	22	3
Family members	22	14	8
Peers	17	7	10
Absent figures	7	2	5

^a^Participants’ texts could be linked to multiple characters (*N* = 25).

Mentors and psychosocial staff appeared across the majorioty of plots as stabilising influences, offering support, guidance, and alternative models of adulthood. Their presence often marked a turning point in participants’ capacity for self-reflection and narrative integration.

Family members were depicted in more conflicted and emotionally complex ways. Some were anchors of affection, especially grandmothers or siblings. Others were a source of profound pain—either due to abandonment, complicity in recruitment, or rejection upon return.

Peers, particularly those who had also exited armed groups, generated belonging and mutual understanding. These connections reduced isolation and provided a safe context in which to explore identity without fear of stigma. However, not all peer relationships were positive. In some cases, peers had played a role in recruitment, contributing to feelings of betrayal and deep mistrust.

Absent characters - including parents, siblings, or recruiters - were often referenced obliquely. These figures shaped internal landscapes but were rarely explored, reflecting unresolved ties. Their absence created gaps that revealed struggles with loss, betrayal, or closure.

### Epiphanies: Turning Points in Narrative Reconstruction

Moments of insight – epiphanies - were critical in shifting how participants understood themselves and their past (Table 7), underpinning increases in self-efficacy —the perceived capacity to act on and shape one’s circumstances (Bandura, 1997). While many participants initially described experiences of powerlessness, over time some began to assert more active roles in decision-making and self-presentation.

**Table 7.** Epiphanies and Their Frequency

Type of epiphany	Participants (N)^a^	Examples
Major	9	Reuniting with a sibling after years of separation, prompting a desire to reconnect with family
		Being offered a scholarship to return to school, sparking a new vision of a future outside violence
		The birth of a child, which inspired a new sense of responsibility and motivation to “be different.”
		Joining a reintegration program where they were called by their real name again for the first time in years
		Being asked to publicly speak at a peace event, helping them see themselves as a voice for change
Minor	7	A psychologist gently correcting their self-description as “a monster,” helping them see themselves as redeemable
		Remembering the taste of a food their grandmother used to make, evoking a lost sense of home and care
		Realising during a workshop that others had experienced similar shame and fear, reducing feelings of isolation
		Being invited to sit at the front of the classroom—an unexpected sign of trust and respect
		Hearing a song that reminded them of childhood, prompting reflection on who they were before the armed group
Cumulative	6	Gradual trust-building with a social worker who consistently showed up and listened without judgment
		Participating in weekly peer dialogue circles where others recognised their emotional growth over time
		Developing a ritual of writing letters to their past self, slowly shifting the tone from blame to compassion
		Receiving repeated feedback from mentors that they were “good at helping others,” shaping a new identity as a helper
		Feeling accepted in a youth group where no one asked about their past, allowing them to feel “normal.”
Re-lived	5	Rewriting the story of a traumatic event through comic panels, transforming horror into reflection and agency
		Drawing a symbolic map of their life and recognising a turning point they had never previously acknowledged
		Performing a theatre piece based on their journey, enabling them to externalise and reinterpret a painful memory
		Listening to another participant’s story and seeing their own past differently in response
		Creating a collage of past and future selves, visually expressing the shift from violence to hope
Dysepiphany	4	Learning that a close friend from the armed group had been killed after demobilisation, reinforcing a sense of futility or survivor’s guilt
		Being publicly identified as a “former guerrillero” during a school event, triggering shame and social rejection
		A failed job interview where the interviewer reacted with fear or disgust after learning about their past

^a^In some cases, participants’ texts could be linked to multiple epiphanies (*N* = 25).

Following Denzin’s (2017) typology, participants described:

Major epiphanies that marked transformative turning points;

Minor epiphanies, where specific memories or realisations helped reframe prior experiences, often linked to safety, care, or unexpected connection;

Cumulative epiphanies, emerging gradually through supportive relationships that fostered self-acceptance and narrative coherence; and

Re-lived epiphanies, enabled through the CAB process, where participants used storytelling or symbolic imagery to reinterpret difficult past events.

These moments catalysed emotional and cognitive shifts and often signalled the beginning of identity reconfiguration (McDonald, 2007). Not all led to growth; some ‘dysepiphanies’ were destabilising, revealing unresolved pain or trauma participants were unprepared to confront (Chappell, 2022).

### Domains of Growth and Repair

Participants revealed diverse patterns of engagement with trauma, identity, and possibility across the four narrative plots. While each plot illustrated a distinct narrative position, cross-case analysis highlighted recurring tensions and shifts in how participants narrated themselves in relation to the past, others, and the future. Six interrelated domains were developed and identified as key to understanding the ongoing process of identity repair among participants:

Narrative coherence refers to the degree to which individuals could organise their experiences into a structured, meaningful story.

Self-concept captures participants’ sense of who they are—their self-image and self-worth—ranging from feelings of shame or damage to emerging self-acceptance.

Self-efficacy reflects their perceived capacity to act on and shape their circumstances and future direction.

Temporal orientation concerns whether their narratives were past-bound, present-focused, or future-oriented.

Relational positioning describes how they positioned themselves in relation to others—whether isolated, mistrustful, connected, or prosocial.

Cognitive processing refers to the narrative work through which participants made sense of their experiences, from intrusive rumination to more deliberate, reflective meaning-making. While more process-oriented than the other domains, it was integral to shifts observed across them (Table 8

**Table 8.** Identity Repair and PTG Among Former Child Soldiers

Domain	Struggler	Learner	Advocate	Survivor	PTG element
Narrative coherence	Fragmented, disjointed	Emerging coherence; competing identities	Goal-driven, socially coherent but sometimes compensatory	Coherent, emotionally regulated, anchored in self-awareness	Existential redefinition
	Fragmented ⇄ integrated				
Self-concept	Shame, self-blame	Developing self-acceptance	Identity based on contribution; potential avoidance	Solidified identity; positive self-perception grounded in endurance	Appreciation of life
	Shame/worthlessness ⇄ self-acceptance				
Self-efficacy	Helplessness, external control	Growing internal agency	High agency focused on helping others	Grounded, autonomous agency; calm self-control	Personal strength
	Powerlessness ⇄ agency				
Temporal orientation	Stuck in past or chaos	Reflective and future-curious	Future-oriented but occasionally idealised	Realistic future orientation, present-motivated	New possibilities
	Past-bound ⇄ future-oriented				
Relational positioning	Isolated, mistrustful	Seeking and rebuilding trust	Seeking connection, beginning trust	Deep relational investment, sometimes at personal cost	Improved relations with others
	Isolation/mistrust ⇄ connection/contribution				
Cognitive processing	Intrusive rumination	Deliberate rumination, emotional processing	Reframing through helping others; possible emotional bypassing	Controlled processing; meaning integrated into worldview	Spiritual/existential change
	Intrusive rumination ⇄ Deliberate rumination				

).

Rather than fixed categories, these domains capture a set of narrative and psychosocial continua - movement through which was often nonlinear, fluid, or contradictory. Relationships, settings, and symbolic resources acted as both anchors and catalysts for change.

## Discussion

The four plots identified offer insight into participants’ shifting self-understandings and the psychosocial conditions shaping their narrative positions. Many entered armed groups having already experienced major disruptions—loss of schooling, unstable caregiving, exposure to violence—that eroded narrative coherence, self-worth, and relational security. On return, these vulnerabilities were compounded by stigma, mistrust, and exclusion from education or work. Identity repair in this context addresses not only conflict-related trauma but also the cumulative erosion of belonging, opportunity, and agency across the life course. For former child soldiers, this is not simply “telling a better story” but actively re-authoring the self through relational dialogue, symbolic expression, and reclaiming disrupted moral and social identities.

Some plots resonate with prior research. The Advocate plot echoes Muldoon et al.’s (2023) concept of collective PTG, where moral agency and prosocial roles support social identity reconstruction. The Struggler plot aligns with Borinca et al.’s (2025) findings that war-related memories and perceived dehumanisation disrupt belonging—though here, such fractures were sometimes repaired through symbolic expression, relational connection, and narrative reframing, often catalysed in CAB workshops.

This typology highlights how reflection, rumination, and epiphanies can reconfigure self-understanding, as supported by research on deliberate rumination (Cann et al., 2011; Durkee et al., 2021). Turning points often arose in liminal spaces where old identities were unsettled but not yet re-stabilised, creating openings for re-authoring the self.

**Figure 1.**
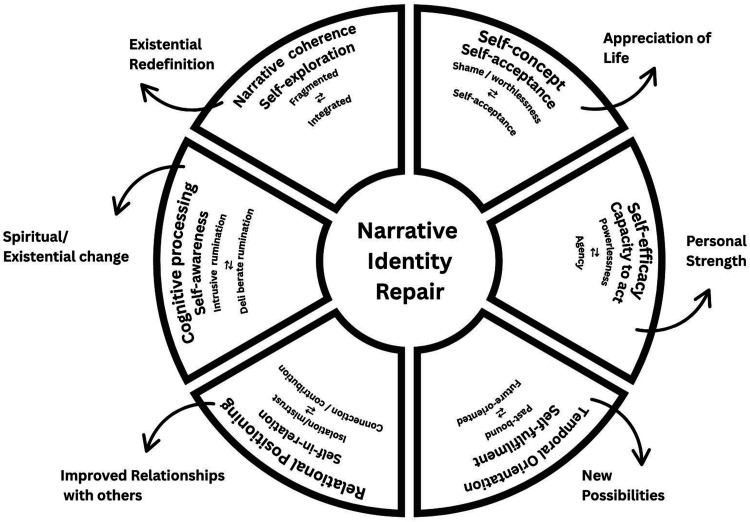
Narrative Repair Compass: Identity repair and PTG

Importantly, these plots represent fluid orientations that participants could move between—or inhabit simultaneously—depending on relational, emotional, and contextual conditions. A young person might articulate a Struggler narrative when reflecting on isolation at home, and an Advocate narrative when describing community activism, with both stories co-existing. These recursive, hybrid positions challenge linear models of development. While identity repair remains a developmental process—shaped by age, memory, reflection, and context—it is not stage-bound. The Narrative Repair Compass (Figure 1) reflects this by mapping growth as dynamic and multidirectional, rather than a singular path to resolution.

The Narrative Repair Compass maps six interconnected domains of self-process each linked to a core element of PTG. It illustrates how identity repair unfolds as a dynamic, relational, and non-linear process, offering direction through the challenges of reintegration after conflict. These domains align with (Wang et al., 2018) self-processes—self-exploration, self-acceptance, self-worth, and fulfilment—while also highlighting the self-in-relation. Within the plots, PTG emerged not as a fixed state but as a fluid, self-constructed possibility shaped by internal and external conditions. This reflects Adler's (2012) finding that narrative coherence and agency language predict well-being over time, and McLean and Syed's (2015) view that repair is socially embedded and culturally shaped.

Unlike PTSD models focused on symptom clusters and clinical recovery, the compass model maps identity repair as relational and developmental. It foregrounds meaning-making, agency, and coherence across six interrelated domains, recognising that change may be recursive, multimodal, and culturally embedded rather than a linear shift from pathology to health.

The model has practical implications for reintegration. For practitioners, recognising a young person’s narrative position is key to tailoring support: Strugglers may require relational safety and trust-building; Learners benefit from structured reflection and skills-building; Advocates need opportunities to channel agency into prosocial action while processing pain; and Survivors may require flexible support to sustain integration. While PTG begins as an internal process, it can be nurtured through consistent support that reinforces agency and connection. Repair is not only about symptom reduction but reclaiming power, rebuilding trust, and reconstructing meaning. From a Power Threat Meaning Framework perspective (Johnstone et al., 2018), this means understanding threats and power imbalances, and the meanings attached to recovery. Relational positioning—how young people see themselves in relation to others—should be a core therapeutic and social goal.

Recent work reinforces the link between social integration and recovery. McMahon et al. (2024) found that social integration mediated the relationship between childhood trauma and physiological stress responses. This underscores the importance of relational and contextual supports, which the present analysis complements by showing how trust, connection, and symbolic recognition shape how youth narrate and enact growth. In practice, reintegration should foster participation, peer connection, and culturally resonant storytelling—conditions that enable both narrative repair and sustainable belonging.

By foregrounding symbolic expression, relational scaffolding, and the coexistence of multiple narrative positions, this study offers a dynamic, contextually grounded understanding of identity repair after armed conflict—acknowledging both its fragility and its potential.

### Limitations and Future Directions

The sample size of this study was small and context-specific, limiting the generalisability of results. Longitudinal research would be needed to understand the sustainability of identity repair and how PTG solidifiess or dissipates over time. It is also important to consider performative dimensions of narrative. In reintegration contexts, growth-oriented accounts may be shaped by cultural scripts or perceived expectations. While the multimodal nature of CAB allowed for indirect expression that could bypass such pressures, social desirability effects cannot be ruled out.

Future research should explore the impact of different support systems on the growth process. There is also a need for comparative studies across post-conflict contexts to better understand how culture and environment shape the expression of PTG as one possible manifestation of repair.

## Data Availability

Due to the nature of the research and to protect the identity of the participants for security reasons, supporting data is not available.*
